# Significance of the Aponeurotic Expansion of the Supraspinatus Tendon in Rotator Cuff Ruptures: An MR Arthrographic Study

**DOI:** 10.3390/medicina62061078

**Published:** 2026-06-02

**Authors:** Rodi Ertogrul, Hayri Ogul, Yusuf Yahsi, Zakir Sakci, Aysenur Dostbil, Mustafa Ozdemir, Mecit Kantarci

**Affiliations:** 1 Department of Orthopedic Surgery, Medical Faculty, Istanbul Medipol University, 34810 Istanbul, Turkey; 2 Department of Radiology, Medical Faculty, Istanbul Medipol University, 34810 Istanbul, Turkey; 3 Department of Radiology, Umraniye Training and Research Hospital, Health Sciences University, 34766 Istanbul, Turkey; 4 Department of Anesthesiology and Reanimation, Ataturk University, 25240 Erzurum, Turkey; 5 Department of Radiology, Medical Faculty, Ataturk University, 25240 Erzurum, Turkey

**Keywords:** supraspinatus tendon, aponeurotic expansion, MR arthrography

## Abstract

*Background and Objectives*: This study aimed to assess the association between the aponeurotic expansion of the supraspinatus tendon (AEST) and supraspinatus tendon tears utilizing magnetic resonance (MR) arthrography in an extensive patient cohort and to determine its clinical relevance. *Materials and Methods*: All MR arthrography images were retrospectively assessed by two radiologists with expertise in arthrography. In both the AEST group and the comparison group consisting entirely of patients with rotator cuff tendon tears, the location and extent of the tear were documented, along with the distance between the tear and the AEST in the study group. In patients with AEST, it was also noted whether the tear extended to or structurally involved the AEST. *Results*: AEST was identified in 61 (7.4%) of the 827 MR arthrograms. The isolated supraspinatus tendon tear emerged as the most prevalent, representing 33.3% of patients with AEST. The triple combination of tears involving the supraspinatus, subscapularis, and infraspinatus accounted for 25.9% of patients with AEST. AEST tears alone were observed in just 3.3% of cases. The incidence of triple combination tears in patients with AEST was found to be significantly lower at 25.9% compared to 59.3% in the control group without AEST (*p* = 0.028). *Conclusions*: In this study, the authors found that the incidence of combined tendon tears in patients with AEST was significantly lower compared to the control group. This result suggests that the AEST likely acts as a structural barrier limiting the anterior progression of rotator cuff tears, particularly toward the subscapularis tendon.

## 1. Introduction

Rotator cuff disorders represent one of the most prevalent causes of shoulder pain and disability, with epidemiological studies demonstrating a strong age-dependent increase in the prevalence of tendinopathy and tendon tearing. The rotator cuff consists of the supraspinatus, infraspinatus, teres minor, and subscapularis tendons, each crucial for maintaining the stability and functionality of the shoulder joint. Among these components, the supraspinatus tendon is most prone to injury and is a leading source of shoulder pain and functional impairment [1]. Rotator cuff tendon tears usually arise from degenerative changes, repetitive microtrauma, and mechanical stress, all of which significantly affect shoulder operation planes [2]. Imaging-based many investigations have reported partial-thickness rotator cuff tears in approximately 13–32% of individuals, whereas full-thickness tears are observed in nearly 10–12% of the general elderly population and may exceed 25% in subjects older than 65 years [3]. Magnetic resonance (MR) imaging studies of asymptomatic volunteers have further demonstrated that rotator cuff abnormalities are frequently incidental findings, with tendinopathy predominating in younger age groups and structural tendon defects becoming increasingly prevalent after the fifth decade of life [4]. In addition, cadaveric and radiological analyses have shown that asymptomatic tears are common, emphasizing the limited correlation between imaging abnormalities and clinical symptoms [5]. These findings collectively support the concept that degenerative tendon changes, aging, and cumulative mechanical loading constitute the principal epidemiological determinants of rotator cuff pathology.

MR arthrography has emerged as a highly reliable imaging modality for the evaluation of rotator cuff tendon tears, particularly in the detection of partial-thickness and articular-sided lesions that may be underestimated on conventional MRI. Recent studies have demonstrated that MR arthrography provides superior sensitivity and diagnostic accuracy for identifying subtle intra-tendinous and footprint abnormalities, with reported sensitivities approaching 90–95% when correlated with arthroscopic findings [6,7]. Furthermore, contemporary comparative analyses have shown that MR arthrography is especially advantageous in the assessment of articular-sided partial-thickness supraspinatus tears and concealed delamination lesions, thereby improving preoperative characterization and surgical planning.

In recent years, the aponeurotic expansion of the supraspinatus tendon (AEST) has been identified as having a significant anatomical relationship with the long head of the biceps tendon (LHBT) [8,9,10]. The influence of AEST on supraspinatus tendon tears is unclear, and only a limited number of studies have addressed this topic in the literature. Jo et al. [11], in their research, suggested that AEST might play a role in the development of tears by altering the load distribution on the supraspinatus tendon. This non-arthrographic MR imaging study demonstrated an association between supraspinatus tendon tears and the presence of AEST. However, researchers of this study have not investigated in detail the key role of AEST on rotator cuff tendon tears. Akkaya et al. reported that no significant correlation was found between presence of AEST and rotator cuff tendon tears [12].

Most existing studies rely on small case series or cadaveric dissections. To our knowledge, no study has systematically evaluated the crucial role of AEST in rotator cuff tendon tears using MR arthrography. Therefore, conducting analyses using MR arthrography will enhance our understanding of the biomechanical impact of AEST on the supraspinatus tendon and its association with tear development. Our theory suggests that in patients with AEST, hypertrophic aponeurosis may prevent extension of the tendon rupture in massive tears of the supraspinatus tendon. The aim of this study was to assess the association between the AEST and supraspinatus tendon tears utilizing MR arthrography in an extensive patient cohort and to establish its clinical relevance. The findings of this study could elucidate the influence of the AEST on supraspinatus tendon tears and enhance our comprehension of this structure’s function in shoulder biomechanics.

## 2. Materials and Methods

### 2.1. Patient Selection

In this retrospective analysis, MR arthrography images from patients referred to our clinic for shoulder MR arthrography between January 2016 and January 2025 were examined in the hospital’s PACS to assess the presence of AEST. This evaluation was conducted by two radiologists with over ten years of experience in musculoskeletal and arthrographic imaging, who reached a diagnosis of AEST through consensus. Out of a total of 850 MR arthrograms, 23 were excluded for various reasons. AEST was identified in 61 (7.4%) of the remaining 827 MR arthrograms. The average age of these 61 patients was 46.25 ± 15.09 years (range = 18–72). Of these patients, 34 (55.7%) were female and 27 (44.3%) were male; 39 patients (63.9%) underwent right shoulder MR arthrography, whereas 22 patients (36.1%) underwent left shoulder MR arthrography. None of the patients underwent bilateral shoulder MR arthrography. Among the 61 MR arthrograms, 27 (44.3%) revealed varying degrees of rotator cuff tendon tears. The extent, size, and affected tendons of these tears in patients with AEST were compared to those in a control group consisting exclusively of individuals with rotator cuff tendon tears. The control group’s mean age was 53.33 ± 10.38 years (range = 27–70). Within this group, 10 patients (37%) were female and 17 (63%) were male, with 19 patients (70.4%) having right shoulder MR arthrography and 8 patients (29.6%) having left shoulder MR arthrography. Ethical approval for the study was obtained from the university’s ethics committee. Patients provided written consent for the MR arthrography procedure.

### 2.2. MR Arthrographic Procedure and Image Analysis

**Figure 1:**
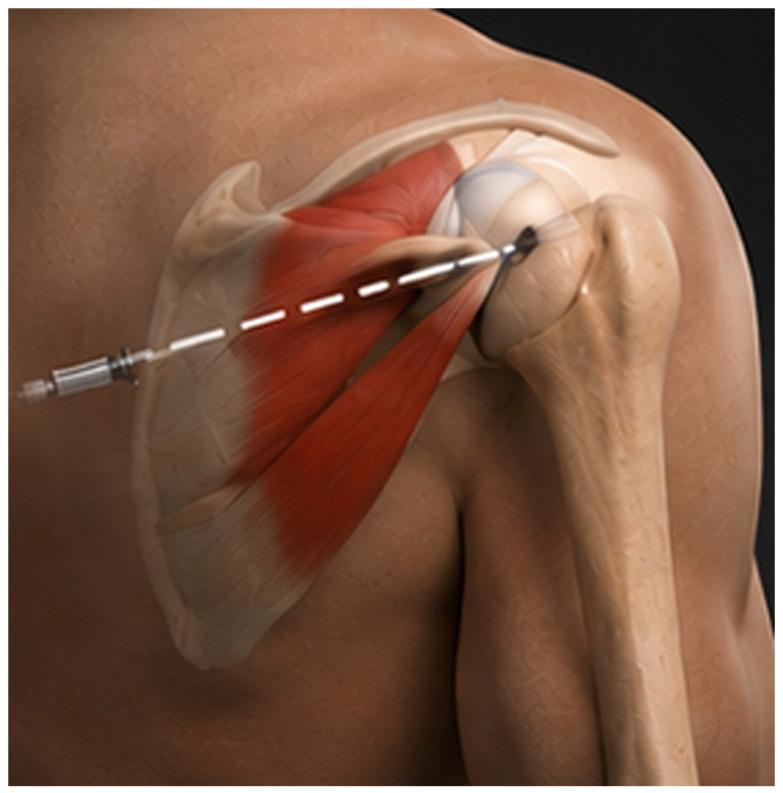
The illustration demonstrates a posterior injection technique for MR arthrography.

In all individuals undergoing MR arthrography, injections were administered via a posterior approach under ultrasound guidance (Figure 1). A 3T MR scanner (the Magnetom Skyra (Siemens Healthcare, Erlangen, Germany) and the MAGNETOM Vida (Siemens Healthineers, Forchheim, Germany)) was employed for capturing the MR arthrographic images. Patients were placed in a supine position on the MR table, and arthrographic images were acquired 15 to 30 min following the injection. Within the shoulder MR arthrography protocol, alongside three-plane fat-suppressed T1 SE MR arthrographic images, a volumetric fat-suppressed T1 MR arthrogram (VIBE = volumetric interpolated breath-hold examination) was obtained, in addition to a thin-slice coronal oblique PD image.

The MR arthrography images were analyzed by consensus of two radiologists. In patients with AEST, the type of aponeurotic expansion, its course adjacent to the bicipital groove, and its proximal attachment pattern were evaluated to assess any association with the presence of a rotator cuff tear. In both the AEST group and the comparison group consisting entirely of patients with rotator cuff tendon tears, the location and extent of the tear were documented, along with the distance between the tear and the AEST in the study group. In patients with AEST, it was also noted whether the tear extended to or structurally involved the AEST.

### 2.3. Statistical Analyses

For statistical analysis, IBM SPSS Statistics 22 (IBM SPSS, Statistics 22, IBM Corp., Armonk, NY, USA) software was utilized. The Shapiro–Wilk test was conducted to assess the normality of parameter distributions. In analyzing the study’s data, descriptive statistical techniques, including mean, standard deviation, and frequency, were employed. One-Way ANOVA was used to compare normally distributed quantitative variables across multiple groups. For parameters not exhibiting a normal distribution, comparisons between two groups were made using the Mann–Whitney U test. In examining qualitative data, the Chi-Square test, Fisher’s Exact test, Fisher-Freeman-Halton test, and Continuity Correction (Yates) were applied. A *p*-value of less than 0.05 was considered statistically significant.

## 3. Results

Research findings reveal that the predominant form of aponeurotic expansion in the supraspinatus tendon is type 1, accounting for 60.7% of cases. Following this, type 2B accounts for 23%, type 2A comprises 13.1%, and type 2C represents 3.3%. The origin area is mainly singular in 86.9% of instances, whereas a smaller subgroup of 13.1% demonstrated a dual origin along with a transverse ligament. Concerning distal insertion regions, a central insertion (between pectoralis major myotendinous junction and pulley region) was present in 47.5% of cases, a myotendinous insertion in 36.1%, and an insertion into the pulley region in 16.4%. Examining the relationship between the AEST and the synovium of the biceps tendon, it was noted that about half (49.2%) were extrasynovial, 26.2% were intrasynovial, and 24.6% displayed both extrasynovial and intrasynovial pathways. When analyzing the AEST pathway in relation to the biceps tendon synovium, the majority was observed in the middle region at 54.1%, with smaller portions located laterally at 16.4% and medially at 23%, while the least amount traversed the entire synovium at 6.6%. Upon assessing pathologies associated with AEST, rotator cuff tears were most frequent at 44.3%, followed by labral pathologies at 32.8%, and biceps pathologies at 13.1% (Table 1, Table 2 and Table 3).

No statistically significant relationships were identified between supraspinatus aponeurosis type and patient age, gender, or shoulder side in this cohort (Table 4, Table 5 and Table 6).

**Figure 2:**
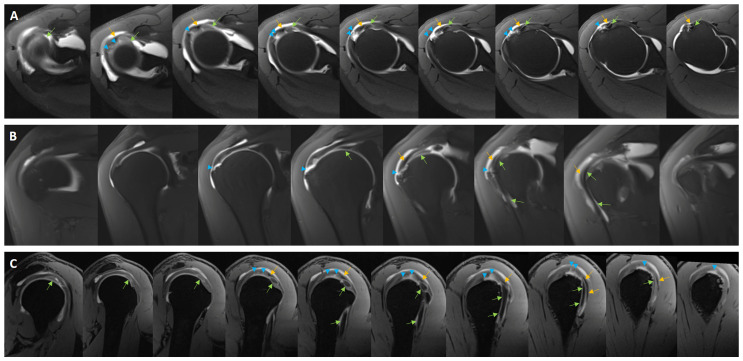
Consecutive axial (**A**), oblique coronal (**B**) and oblique sagittal (**C**) T1-weighted MR arthrograms reveal association between AEST and rotator cuff tear. The consecutive images show partial full thickness tear of the supraspinatus tendon without tear of the subscapularis tendon and AEST. Blue arrowheads = supraspinatus tendon tear; yellow arrows = AEST; green arrows = LHBT.

Upon analyzing rotator cuff tears associated with AEST, the isolated supraspinatus tendon tear emerged as the most prevalent, representing 33.3% of cases (Figure 2). This was succeeded by the triple combination of tears involving the supraspinatus, subscapularis, and infraspinatus, accounting for 25.9%. Isolated subscapularis tears were observed in only 7.4% of instances. Tears involving both the supraspinatus and subscapularis were identified in 11.1% of cases, while those involving the supraspinatus and infraspinatus constituted 22.2%. AEST tears alone were observed in just 3.3% of cases. Within the cohort of AEST patients, the average size of the rotator cuff tendon tear, beginning from the supraspinatus tendon and extending to the teres minor tendon, was calculated to be 20.8 ± 12.37 mm (range: 4–52 mm). The mean distance between starting point of the rotator cuff tear and AEST was measured at 1.88 ± 4.26 mm (range: 0–15 mm).

**Figure 3:**
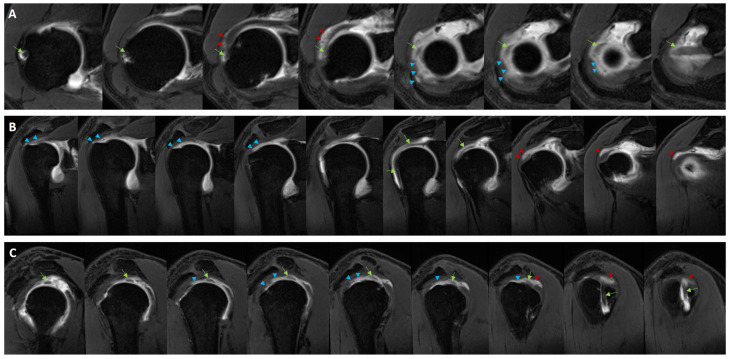
Consecutive axial (**A**), oblique coronal (**B**) and oblique sagittal (**C**) T1-weighted MR arthrograms reveal extending into the subscapularis tendon via the transverse humeral ligament of the supraspinatus and infraspinatus tendon tear without AEST. Blue arrowheads = supraspinatus and infraspinatus tendon tear; red arrowheads = subscapularis tendon tear; green arrows = LHBT.

In the comparison cohort, composed entirely of individuals with rotator cuff tears and devoid of AEST, the average dimension of the rotator cuff tear was recorded as 23.59 ± 11.84 mm, with a range from 5 to 41 mm. In this group, which demonstrated a 100% occurrence of rotator cuff tears, the most frequently associated lesion was biceps tendon pathology, present in 40.7% of cases, followed by labral pathology, which appeared in 37% of cases. Within this cohort, the most prevalent type of rotator cuff tear identified was the triple combination involving the supraspinatus, subscapularis, and infraspinatus tendons, observed in 59.3% of cases (Figure 3). Isolated supraspinatus tears were identified in only 18.5% of cases. Tears involving both the supraspinatus and subscapularis tendons were present in 14.8% of cases, whereas those affecting the supraspinatus and infraspinatus appeared in 7.4% of cases. No instances of isolated subscapularis tendon tears were reported.

An analysis of the AEST type, its synovial pathway, and its connection with the synovium demonstrated no statistically significant correlation with the occurrence of rotator cuff tears (*p* = 0.745, *p* = 0.434, *p* = 0.642; *p* > 0.05, respectively).

A statistically significant correlation was not identified between the incidence of rotator cuff tendon tears and the supraspinatus tendon having a single origin from the AEST or having dual origins from both the supraspinatus tendon and the transverse ligament (*p* = 0.067; *p* > 0.05).

There was no statistically significant difference in the size of rotator cuff tears between patients with AEST and those in the control group without AEST (*p* = 0.263; *p* > 0.05).

No statistically significant difference was identified between the groups with and without AEST concerning the prevalence of isolated supraspinatus tendon tears, combined supraspinatus and subscapularis tendon tears, and combined supraspinatus and infraspinatus tendon tears (respectively, *p* = 0.352, *p* = 0.500, *p* = 0.125; *p* > 0.05).

The incidence of combined tendon tears involving the supraspinatus, subscapularis, and infraspinatus in patients with AEST was found to be statistically significantly lower at 25.9% compared to 59.3% in the control group without AEST (*p* = 0.028; *p* < 0.05).

The results of this study demonstrate that the AEST is a common but often overlooked variation in shoulder anatomy and may have a potential biomechanical effect on the progression pattern of rotator cuff pathologies. Specifically, the significantly lower incidence of massive combined rotator cuff tears (supraspinatus-subscapularis-infraspinatus combination) in the presence of AEST suggests that this structure may act as a limiting or protective barrier against the progression of tendon tears. Clinically, these findings indicate that recognizing AEST as a normal anatomical variation in patients with shoulder pain and rotator cuff pathology can prevent misdiagnosis. For radiologists, understanding the relationship of AEST to the synovium and its different course types can prevent confusion with partial tears, synovitis, or pathological fibrous bands in MR arthrography and shoulder MRI scans. For surgeons, knowing the anatomical localization of the AEST in arthroscopic procedures can facilitate surgical planning, especially around the biceps pulley region and rotator ridge, and reduce the risk of unnecessary debridement or iatrogenic damage.

## 4. Discussion

**Figure 4:**
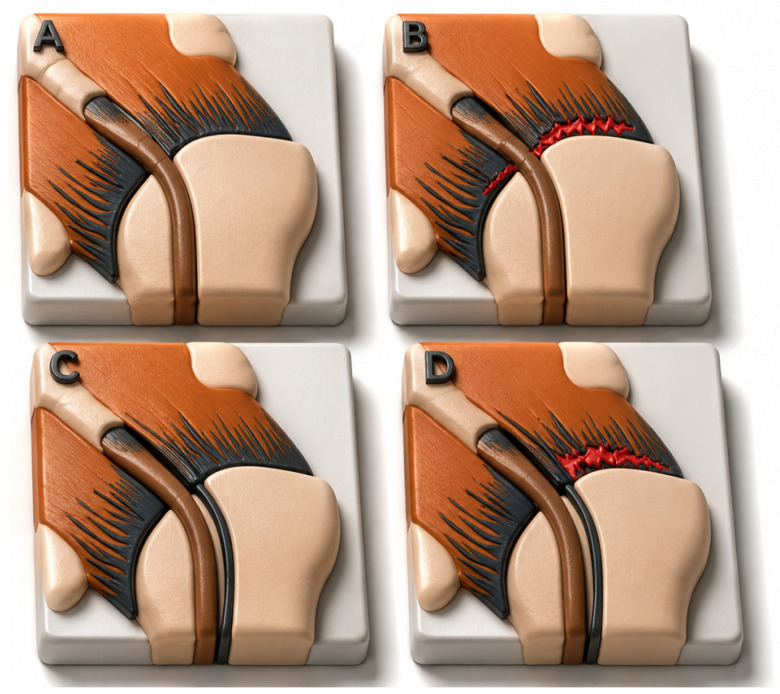
Illustration demonstrating anterior extension of the rotator cuff tear without AEST (**A**,**B**) and the AEST preventing the extension of the tear (**C**,**D**).

This study investigated the potential restricting role of the AEST in impeding the anterior extension of rotator cuff tears, particularly concerning the subscapularis tendon. In a review of 827 MR arthrography examinations, AEST was found in 61 patients, of whom 27 exhibited rotator cuff tears. A comparison was drawn with 27 control patients close in age who had rotator cuff tears without the presence of AEST. The results demonstrated that the control group, which lacked AEST, exhibited a significantly higher occurrence of massive tears (59.3% vs. 25.9%, *p* = 0.028) and more frequent involvement of the subscapularis tendon. These findings suggest that AEST may serve as a structural barrier, preventing the expansion of rotator cuff tears toward the subscapularis tendon (Figure 4).

In this research, MR arthrography was utilized as the primary imaging technique due to its superior sensitivity over conventional MR imaging in assessing subtle anatomical variations, such as AEST [13]. The application of intra-articular contrast facilitated precise delineation of the localization, morphological subtype, and anatomical relationships of AEST with surrounding tendons. Additionally, the enhanced visualization of the direction of tear propagation significantly strengthened the diagnostic capability of the study.

Although the AEST has been thoroughly explored in anatomical texts for a considerable period, its functional importance has only recently become a focal point of investigation. Moser and colleagues have clarified that this anatomical structure originates from the anterior and superficial fibers of the supraspinatus tendon, extending toward the superior margin of the pectoralis major tendon [8]. In terms of histological analysis, Clark and Harryman have described the aponeurotic extension as arising from the superficial layer of the supraspinatus, encircling the biceps tendon and forming a connection between the rotator cuff and anterior shoulder components [14]. Various morphological types of AEST have been identified, with imaging studies suggesting its prevalence ranges from 20% to 50% [8,11].

The hypothesis that AEST may act as a biomechanical barrier against the propagation of rotator cuff tears is of interest from both anatomical and functional perspectives; however, the current literature does not provide robust and direct evidence to support this concept. Nevertheless, given the critical role of intra-tendon load distribution and local stress concentrations in rupture progression, this structure can function as a secondary stabilizing element capable of redistributing mechanical stress. Previous biomechanical studies have shown that the direction of propagation of a rotator cuff tear is largely influenced by increased major stress zones within the tendon, particularly in the anterior supraspinatus fibers, and that increased stress in these zones can facilitate posterior extension of the tear [15]. Furthermore, finite element-based studies have shown that the location and thickness of the tear significantly alter intra-tendonal stress patterns [16]. In this context, AEST, which maintains continuity with the fibrillar architecture of the supraspinatus tendon, could theoretically regulate load transfer in the anterior-superior region and thus partially limit tear propagation. However, because this potential protective role has not yet been directly demonstrated through dedicated biomechanical investigations, the hypothesis remains largely speculative.

Aponeurotic expansion can also play an important role in distributing the load on the rotator cuff in patients who have undergone mass surgery and prosthesis implantation in the proximal humerus [17,18]. The authors of a study by Akkaya and colleagues [12] considered AEST not merely as an anatomical variation, but as a functional component of the rotator cuff complex. Their findings suggested that this structure demonstrates close anatomical continuity with the long head of the biceps tendon and surrounding rotator cuff fibers, can be reliably identified on MRI, and may contribute to preservation of shoulder function even in the presence of advanced tendon pathology. However, because the study was retrospective and primarily imaging-based, lacking histological confirmation and dynamic biomechanical analysis, it cannot directly establish that the aponeurotic expansion functions as a true “protective barrier.” Moreover, the authors themselves did not definitively clarify whether this structure facilitates tear formation or instead represents a compensatory mechanism aimed at maintaining tendon function. Despite these limitations, the study remains valuable because it integrates tendon biology with imaging correlations and highlights that the supraspinatus tendon possesses a far more complex stress-transfer network than previously assumed under the traditional concept of a homogeneous tendon structure.

Similarly, the study associated with demonstrated that rotator cuff tear behavior cannot be fully explained solely by macroscopic tear size, emphasizing instead the importance of regional mechanical properties and structural heterogeneity within the tendon [2]. The authors showed that the anterior supraspinatus fibers exhibit distinct strain characteristics and that tear propagation tends to follow specific mechanical stress vectors. These findings indirectly support the hypothesis that an AEST may reduce focal stress concentration and thereby influence tear progression; however, the available evidence remains insufficient to confirm that it acts as an independent biomechanical barrier. Contemporary insights into tendon biology indicate that degenerative rotator cuff disease is primarily driven by collagen disorganization, impaired cellular mechanotransduction, and weakening of the enthesis microstructure. Therefore, the presence of an aponeurotic expansion should perhaps be interpreted not as an isolated protective factor, but rather as part of a broader compensatory remodeling response within the tendon. Future investigations combining high-resolution imaging, histopathological assessment, and in vivo biomechanical loading models will be essential to determine whether this structure truly exerts a clinically meaningful stress-shielding effect during rotator cuff tear progression.

The functional role of this anatomical structure aligns with the typical biomechanical attributes of aponeuroses within the muscle–tendon complex. Wheatley et al. highlighted that aponeurotic tissues are crucial for load distribution and force transmission, with their heterogeneous composition enhancing mechanical resilience [19]. In this framework, AEST might facilitate force transfer within the rotator cuff, thereby mitigating tear propagation. Notably, our research indicated a significant correlation between the presence of AEST and reduced anterior tear extension, along with a decreased incidence of subscapularis involvement. Absent AEST’s structural support, one would not anticipate a significant disparity between groups; nonetheless, our results clearly contradict this assumption.

The hypothesis receives further validation from a case study documented by Uludağ et al., where a patient with a significant tear demonstrated preservation of the AEST and exhibited shoulder functionality surpassing expectations. In this initially noted case, the AEST remained intact despite the presence of massive tear of the supraspinatus and infraspinatus tendons, while the subscapularis tendon had also a slight partial tear [10]. These findings suggest that the AEST functions not only as an anatomical structure but also as a biomechanical barrier.

It is widely acknowledged that rotator cuff tears generally extend in the posterosuperior direction, affecting the supraspinatus and infraspinatus tendons, while the anterosuperior extension, which includes the subscapularis, is less prevalent. Prior studies have shown that this anterior progression is constrained by anatomical structures such as the rotator interval and the biceps pulley [20,21,22]. The AEST may collaborate with these structures to establish the anterior limit of tear expansion.

Our research provides a novel contribution to the existing literature. To our knowledge, this is among the first controlled case series to demonstrate a direct correlation between the presence of AEST and the patterns of tear propagation. From these findings, we suggest that AEST might aid in preventing the anterior progression of supraspinatus tears toward the subscapularis tendon. Simultaneously, it may uphold the functional integrity of the rotator cuff by facilitating intertendinous force transmission.

This research is subject to several limitations. The retrospective nature of the study precluded the standardization of MR imaging parameters. The control group was drawn not from healthy individuals but from a patient database comprising those with rotator cuff tears. This situation leads to significant selection bias and prevents a meaningful assessment of the prevalence of AEST in the general population. Furthermore, some cases did not have arthroscopic confirmation, and no biomechanical analysis was conducted. Consequently, future research should adopt a prospective design, incorporate arthroscopic validation, employ cadaveric studies, and conduct biomechanical assessments to furnish more robust evidence supporting our hypothesis.

## 5. Conclusions

In summary, our investigation suggests that the AEST likely acts as a structural barrier that limits the anterior progression of rotator cuff tears, particularly toward the subscapularis tendon. This function may consequently reduce the incidence of extensive tears. The presence of AEST should be interpreted not as an isolated protective factor, but as part of a broader compensatory remodeling response within the tendon. Future research combining high-resolution imaging, histopathological evaluation, and in vivo biomechanical loading models is crucial to determine whether this structure exhibits a clinically significant stress shielding effect during rotator cuff tear progression.

## Figures and Tables

**Table 1:** Distribution of AEST Types.

Type	Percentage (%)
Type 1	60.7
Type 2B	23.0
Type 2A	13.1
Type 2C	3.3

**Table 2:** Comparison of Rotator Cuff Tear Patterns.

Tear Pattern	AEST Group (%)	Control Group (%)
Isolated SSP	33.3	18.5
SSP + SSC + ISP	25.9	59.3
Isolated SSC	7.4	0.0
SSP + SSC	11.1	14.8
SSP + ISP	22.2	7.4

**Table 3:** Associated Pathologies.

Pathology	AEST (%)	Control (%)
Rotator cuff tear	44.3	100.0
Labral pathology	32.8	37.0
Biceps pathology	13.1	40.7

**Table 4:** Age Distribution According to Aponeurosis Type.

Aponeurosis Type	n	Mean Age	SD
TYPE 1	37	44.70	15.83
TYPE 2A	8	49.88	15.34
TYPE 2B	14	46.00	13.12
TYPE 2C	2	62.00	8.49

One-way ANOVA showed no statistically significant difference in age among aponeurosis types (F = 1.01, *p* = 0.394).

**Table 5:** Gender Distribution According to Aponeurosis Type.

Aponeurosis Type	Female	Male
TYPE 1	20	17
TYPE 2A	3	5
TYPE 2B	9	5
TYPE 2C	2	0

Chi-square analysis demonstrated no significant association between aponeurosis type and gender (χ^2^ = 3.12, *p* = 0.373).

**Table 6:** Shoulder Side Distribution According to Aponeurosis Type.

Aponeurosis Type	Left	Right
TYPE 1	12	25
TYPE 2A	3	5
TYPE 2B	6	8
TYPE 2C	1	1

Chi-square analysis demonstrated no significant association between aponeurosis type and shoulder side (χ^2^ = 0.67, *p* = 0.881).

## Data Availability

The data presented in this study are available on request from the corresponding author due to reasons of sensitivity of human data.

## Abbreviations

AEST	the aponeurotic expansion of the supraspinatus tendon
LHBT	the long head of the biceps tendon
MR	magnetic resonance
VIBE	volumetric interpolated breath-hold examination
